# Sir Percivall Pott: A Stalwart Contributor to the World of Surgery

**DOI:** 10.7759/cureus.78663

**Published:** 2025-02-07

**Authors:** Jagdish U Patil, Mukesh O Phalak, Ajinkya K Chaudhari, Tushar Chaudhari

**Affiliations:** 1 Department of Orthopaedics, Dr. D. Y. Patil Medical College, Hospital, and Research Centre, Dr. D. Y. Patil Vidyapeeth (Deemed to be University), Pune, IND

**Keywords:** biographical memoir, historical vignette, medical pioneer, percivall pott, pott, sir percivall pott

## Abstract

Sir Percivall Pott (1714-1788) was a prominent English surgeon renowned for his substantial contributions to surgery and orthopedics. His career began with the preparation of cadavers for dissection under Edward Nourse, where he studied anatomy. Among his many significant contributions, Pott advocated for limb-preserving techniques over radical methods such as amputation. He was the first to identify an environmental carcinogen and link it to cancer. His well-known account of sustaining a bimalleolar ankle fracture from a horse fall, which was managed conservatively without any residual deformity and later became known as Pott's fracture. Additionally, his research on spinal tuberculosis, now referred to as Pott's disease, is widely recognized in the medical community. Pott authored numerous publications that encompass both surgical and orthopedic advancements. This review aims to pay tribute to this eminent figure, highlighting his groundbreaking discoveries that have laid the foundation for several concepts in surgical sciences.

## Introduction and background

Sir Percivall Pott (6^th^ January 1714 to 22^nd^ December 1788), an English surgeon, occupies a distinguished place in the annals of medical history. He is known for many insightful and comprehensive surgical writings. His pioneering work in surgery, especially in the treatment of fractures, spinal tuberculosis, and occupational health, has left an indelible mark on modern medicine. Groundbreaking discoveries starting from associating scrotal cancer with soot (an environmental carcinogen) in chimney workers to significant contributions in orthopedics in ankle fractures and tuberculosis of the spine make this gentleman a noteworthy contributor whose work impacts modern-day evidence-based medicine. This review pays tribute to Pott’s contributions and delves deeply into Pott's life and the enduring legacy of his work. It aims to provide a thorough understanding of how Pott's innovations have shaped medical practice and public health.

## Review

Early life and medical training

Percivall Pott was born on January 6, 1714, in Threadneedle Street, England, to Elizabeth Pott and Percivall Pott Senior [1]. His father’s untimely demise when Pott was a child left his mother with the responsibility of raising Pott. Joseph Wilcocks, a relative of his mother, financed the education of Pott. He was sent to a private school in Darenth, Kent. Despite these challenges, Pott exhibited a remarkable aptitude for learning and a keen interest in medicine from a young age. He had learned Latin and Greek in school and had an interest in classical cultures in school. His initial exposure to the medical field came through an apprenticeship with Edward Nourse, a local surgeon, a common practice in 18th-century England that combined practical experience with rudimentary theoretical training. Pott used to prepare subjects for anatomy and surgery demonstrations for Edward Nourse [1]. His apprenticeship was for seven years, which helped Pott provide for his family. Pott then appeared before the court of examiners of the Company of Barber-Surgeons and proved himself, allowing him to practice [2]. In 1745, he was appointed as an assistant at St. Bartholomew’s Hospital and later was promoted to surgeon at the hospital [3]. He studied under eminent surgeons of the time and was influenced by the burgeoning scientific approach to medicine. The hospital provided a rich environment for clinical practice, allowing Pott to gain hands-on experience in treating various conditions, which would later inform his groundbreaking work.

Professional career and innovations

**Figure 1:**
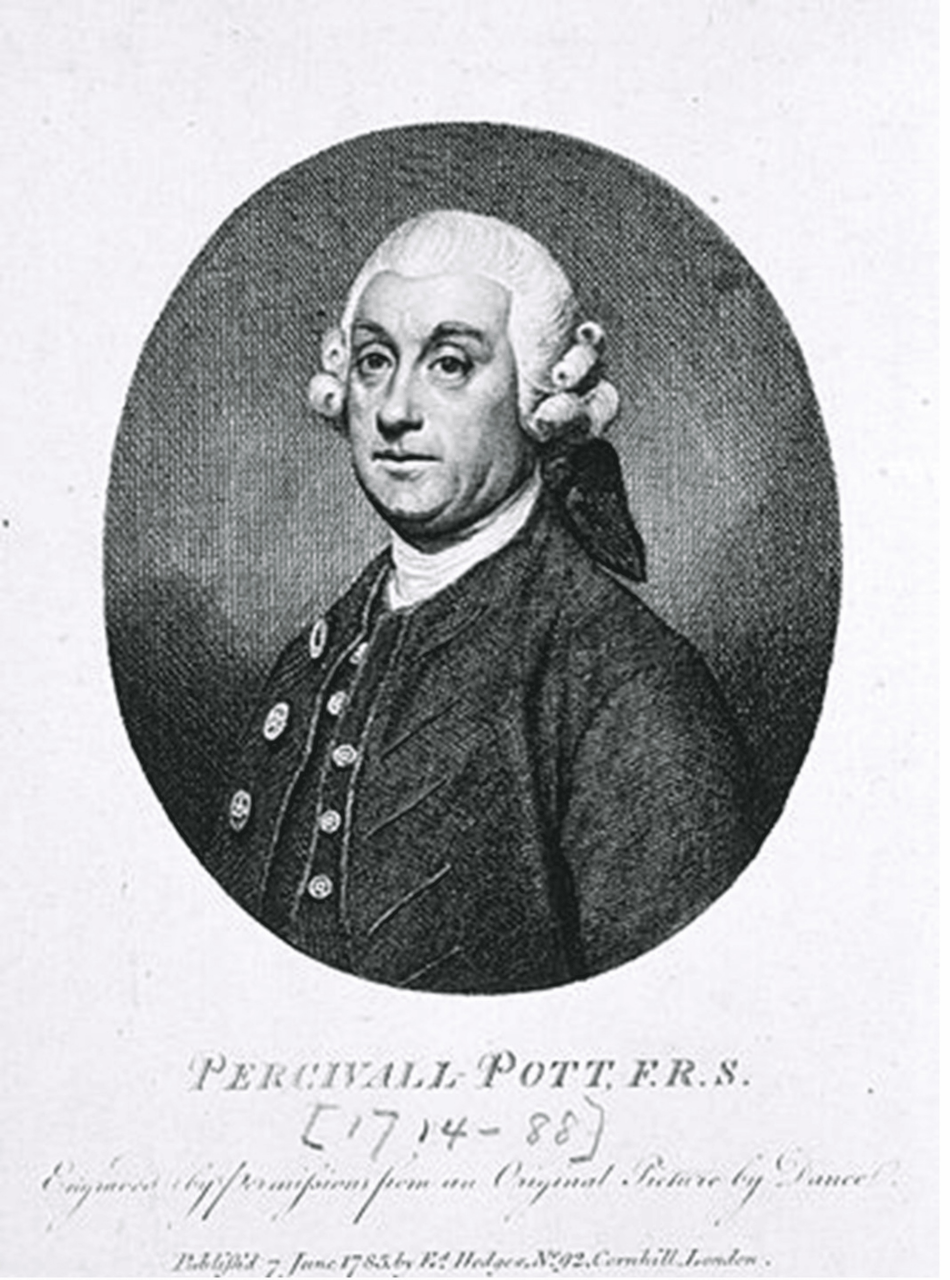
Percivall Pott, engraved from an original picture by Nathaniel Dance-Holland Source: National Library of Medicine [4]. This image is in the public domain.

In 1745, Pott married Sarah Creuttenden and moved to Watling Street, where he was appointed surgeon. In 1753, Pott was elected Master of Anatomy at the Surgeons’ Hall. In 1756, he had a fall from his horse during his visit to the Anatomy School in Covent Garden and sustained an ankle fracture. He, against advice, did not opt for amputation and survived the incident and wrote his famous Treatise on Ruptures. In 1756, Pott joined the Court of Assistants of the Surgeons' Company; in 1761, he was elected to the Court of Examiners; and in 1765, he was elected as the Master (Figure 1) [1, 4]. He then moved to Princes Street in 1769 and enjoyed practicing in London [1]. In 1788, he had traveled 20 miles to see a patient in harsh weather, owing to which he caught a cold, fever, chills, and delirium, ultimately leading to the end of a legend. The dedication and conviction to patient care exhibited by Pott is an example to be remembered even today [3]. Pott received recognition for his work in the form of various honors summed up in Table 1.

**Table 1:** Honours received by Sir Percivall Pott

Honours Received by Sir Percivall Pott	
1765	Election to Fellowship of the Royal Society
1786	Honorary Diploma of the Royal College of Surgeons of Edinburgh
1787	Freedom of the Irish College

Surgical techniques

Pott's career as a surgeon was characterized by a series of innovative techniques and improvements. He used to advocate against the use of blind cautery and is said to have proposed simple instrumentation. He used to advise young surgeons to develop dexterity in their practice and not run behind the mere speed of the surgery [1]. He also emphasized the importance of focusing on patient management and pain alleviation rather than surgical intervention. In his papers, he cautioned the surgeons against the use of amputation and discouraged his fellow surgeons from such drastic surgeries.

Pott also made substantial contributions to the management of surgical wounds. He was an early advocate for cleanliness and antisepsis in surgical practice, understanding the importance of preventing infection through meticulous hygiene and proper wound care. His emphasis on these practices contributed to a reduction in postoperative infections and set a standard for surgical procedures. In 1745, he used to give lectures to the dressers of the hospital to make them better at wound management [1].

Fracture management

**Figure 2:**
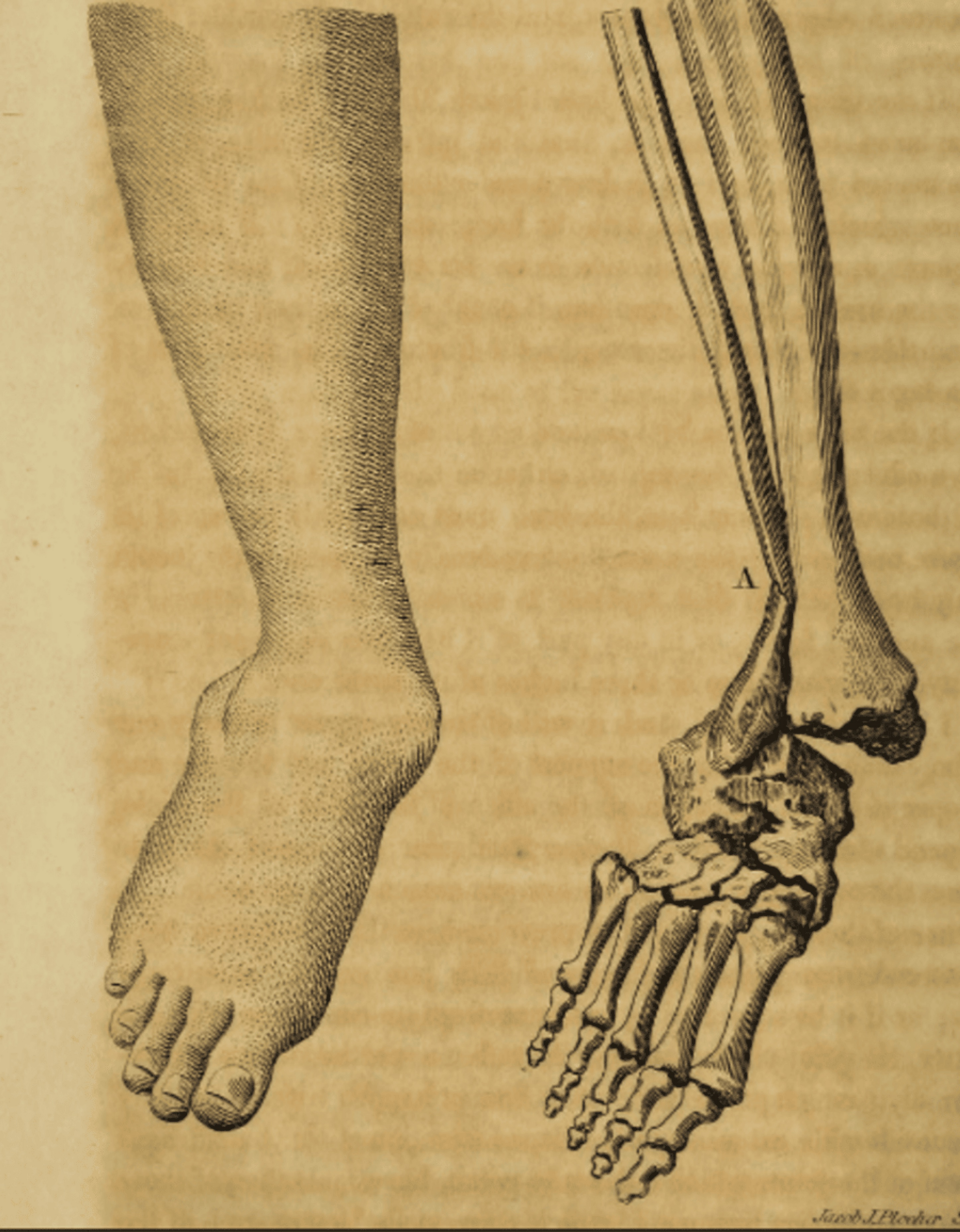
Pott's fracture This image shows the clinical deformity of the fracture at the ankle joint and the diagrammatic depiction of the fracture. Source: [7]; Public Domain Mark 1.0 Universal

One of his most significant contributions was in the treatment of fractures. In 1756, when Pott sustained a compound ankle fracture by falling from horseback, he applied his knowledge and did not get up from the ground. Instead, he asked his assistant to buy a door and used it as a stretcher. He immobilized his leg and traveled to avoid further damage [5]. His mentor, Edward Nourse, advocated against amputation and advised reducing the bone and managing it conservatively. Later, this fracture of the ankle came to be known as Pott’s fracture in his memory (Figure 2) [5,6,7]. Before Pott's advancements, fracture management was rudimentary and often led to poor outcomes. Pott disagreed with the drastic surgical procedures. In 1765, Pott, through his paper on fractures and dislocations, introduced the importance of immobilizing broken bones, a method that drastically improved healing rates and patient outcomes. His approach emphasized the importance of immobilization and proper alignment, principles that remain fundamental in modern orthopedic practice [8].

Pott's disease

Among Pott's most significant contributions was his work on spinal tuberculosis, now known as Pott's disease. In the 18^th^ century, tuberculosis was a poorly understood and often fatal disease. Pott's clinical observations led him to recognize that tuberculosis could affect the spine, causing severe deformities and debilitating pain.

**Figure 3:**
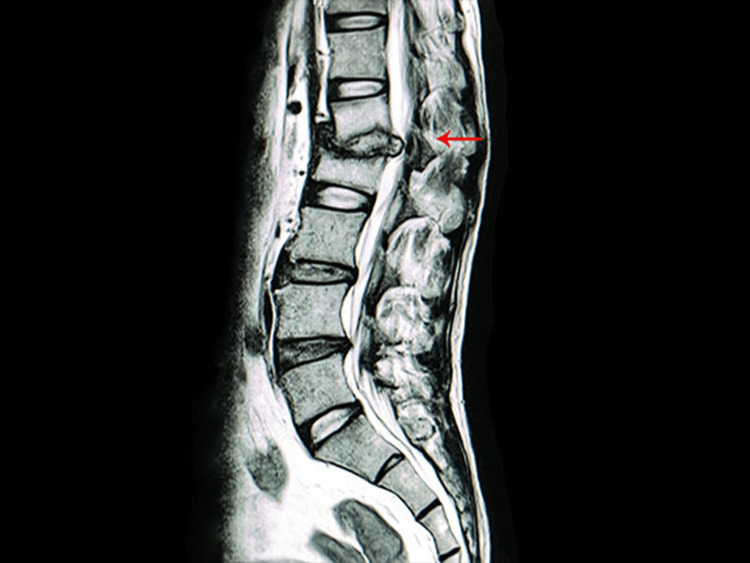
An MRI depicting tuberculosis of the spine T2 weighted sagittal MRI cut of the lumbosacral spine, red arrow depicting discal and paradiscal involvement of the L1-L2 disc and endplates of L1 and L2 with compression of posterior neural structures most probably due to infective etiology due to tuberculosis of the spine (Pott's spine) This image is from the authors' institution.

In his seminal work, *Chirurgical Observations* (1779), Pott meticulously described the symptoms and progression of spinal tuberculosis. He identified the disease's characteristic features, such as spinal deformity, paralysis, and abscess formation, and advocated for early diagnosis and intervention [9]. Figure 3 is an example of this. Pott's observations were groundbreaking in an era when the connection between tuberculosis and spinal disease was not well established. His work laid the foundation for future research and treatment strategies for spinal tuberculosis.

Scrotal cancer in chimney sweepers

Pott's insights extended beyond traditional surgical practices to the emerging field of occupational health. In a pioneering study of chimney sweeps, Pott observed a high incidence of scrotal cancer among these workers, who were exposed to soot and other carcinogenic substances. This observation was one of the earliest documented cases linking occupational exposure to cancer. In a paper in 1775, he regarded it as chimney sweepers’ cancer. He suggested the removal of the affected scrotal sore to prevent the spread of cancer to the testes, which could not be controlled even by castration [10].

Pott's research in this area was crucial in establishing the concept of occupational health risks and the need for protective measures. His work highlighted the importance of recognizing and mitigating health hazards associated with various occupations, leading to the development of health and safety regulations designed to protect workers from harmful exposures. This aspect of Pott's work has had a profound impact on public health policies and occupational safety standards.

Legacy of Sir Percivall Pott

Pott has summed up his learning in the form of various books and publications for the subsequent generations to come, his contributions are summarized in Table 2 [11-22].

**Table 2:** Publications by Sir Percivall Pott

Publications and contributions by Sir Percivall Pott	
1741	An account of tumours which rendered the bones soft [11]
1756	A treatise on ruptures (2^nd^ edition : 1763; 3^rd^ edition: 1769; 4^th^ edition: 1775) [12]
1757	An account of a particular kind of rupture, frequently attendant upon newborn children; and sometimes met with in adults; viz. that in which the intestine or omentum is found in the same cavity, and in contact with the testicle [1,13]
1758	Observations on that disorder of the corner of the eye, commonly called fistula lachrymalis (2^nd^ edition: 1763; 3^rd^ edition: 1769; 4^th^ edition: 1772; 5^th^ edition: 1775) [14]
1760	Observations on the nature and consequences of wounds and contusions of the head, fractures of the skull, concussion of the brain [15]
1762	Practical remarks on the hydrocele or watery rupture, and some other diseases of the testicle, its coats, and vessels; illustrated with cases; being a supplement to a general treatise on ruptures, published in the year 1756 [1]
1765	An account of a hernia of the urinary bladder, including a stone [16]
1765	Remarks on the disease, commonly called a fistula in ano (2^nd^ edition: 1767; 3^rd^ edition: 1771; 4^th^ edition: 1775) [17]
1768	Observations on the nature and consequences of those injuries to which the head is liable from external violence [18]
1768	Some few general remarks on fractures and dislocations [19]
1771	An account of the method of obtaining a perfect or radical cure of the hydrocele, or watery rupture, by means of a seton [1]
1775	Chirurgical observations relative to the cataract, the polypus of the nose, the cancer of the scrotum, the different kinds of ruptures, and the mortification of the toes and feet [20]
1779	Remarks on that kind of palsy of the lower limbs, which is frequently found to accompany a curvature of the spine, and is supposed to be caused by it together with its method of cure; Remarks on that kind of palsy of the lower limbs which is frequently found to accompany a curvature of the spine [21]
1782	Further remarks on the useless state of the lower limbs, in consequence of a curvature of the spine [22]

Impact on modern health practices

Sir Percivall Pott's contributions to both surgery and public health had a lasting and transformative effect on modern practices. His work has been widely studied and referenced in medical education, and his principles are incorporated into medical curricula and textbooks. As one of the leading surgeons of his time, Pott was instrumental in advancing the field of surgical techniques, especially in treating fractures and managing complex injuries. He introduced a methodical approach to surgical procedures, emphasizing the importance of detailed anatomical knowledge and precise techniques. His techniques for managing fractures and treating wounds have been refined and expanded upon in modern medicine. The principles of immobilization are integral to current surgical practices. His work on fractures, including the use of splints and the treatment of spinal injuries, provided a foundation for modern orthopedics. His work paved the way for the development of advanced orthopedic techniques and contributed to the evolution of surgical standards.

In public health, Pott is perhaps best known for his groundbreaking work in occupational health and for being instrumental in shaping modern occupational health practices. His study of scrotal cancer in chimney sweeps in the 18^th^ century, where he linked the disease to prolonged exposure to soot, marked one of the first documented instances of occupational cancer. This discovery was revolutionary, as it highlighted the direct connection between environmental factors and disease, particularly the harmful effects of workplace conditions on long-term health. Pott’s findings led to public health reforms, such as the regulation of chimney sweeping practices, which were intended to reduce exposure to hazardous substances and improve the safety of laborers. His work on occupational diseases helped inspire the establishment of more comprehensive public health policies and contributed to the growing recognition of the need to protect workers from environmental hazards. His observations have led to the implementation of safety measures and regulations that protect workers from exposure to harmful substances. Pott's legacy in this area continues to influence public health policies and workplace safety standards.

Pott’s influence extended beyond just the field of surgery and public health. His methods promoted a scientific, evidence-based approach to medicine that laid the groundwork for later advancements in epidemiology, the study of how diseases spread within populations. By emphasizing observation, meticulous record-keeping, and a systematic approach to medical problems, Pott helped shape the modern approach to diagnosing, preventing, and treating diseases, particularly those linked to environmental or societal factors. His pioneering work in these areas continues to inform both surgical practices and public health policies today. His contributions have shaped the development of medical education and practice, ensuring that his influence endures in the field.

## Conclusions

Sir Percivall Pott was a stalwart whose contributions to medicine and surgery have had a lasting impact. His contributions in surgical techniques, his groundbreaking work on spinal tuberculosis, and his insights into occupational health have shaped modern medical practice and public health policies. Pott's emphasis on empirical observation, cleanliness, and patient care set new standards in the medical field and continue to influence modern-day medicine.

As we reflect on Pott's achievements, it is evident that his work has left an indelible mark on the history of medicine. We give a tribute to his legacy, which endures in the principles of surgical practice, occupational safety, and medical education. Sir Percivall Pott's life and work are a testament to the enduring quest for knowledge and the relentless pursuit of better health for all.
